# The Italian Version of the Five-Word Test: A Simple Diagnostic Test for Dementia due to Alzheimer's Disease in Routine Clinical Practice

**DOI:** 10.1155/2017/3781407

**Published:** 2017-09-25

**Authors:** Luca Rozzini, Anna Ceraso, Marina Zanetti, Silvia Pelizzari, Evita Tomasoni, Vivian Accardo, Alessandro Padovani

**Affiliations:** Department of Clinical and Experimental Sciences, University of Brescia, Piazzale Spedali Civili 1, 25123 Brescia, Italy

## Abstract

**Background:**

The five-word test (FWT) is a neuropsychological tool (derived from the Grober and Buschke paradigm), measuring hippocampal memory trace consolidation. The study aimed to validate the test for the Italian language and to verify its ability to discriminate patients affected by mild cognitive impairment and dementia due to Alzheimer's disease from healthy matches.

**Methods:**

217 subjects (127 controls, 47 MCI due to AD, and 43 AD) underwent neuropsychological evaluation. The Spearman rank coefficient (*ρ*) was used to assess the correlation between immediate (IRS), delayed (DRS), and total score (TRS) of the FWT and correspondent matches of a specific short story test, while receiving operator characteristic (ROC) curves were built to investigate the diagnostic accuracy of both.

**Results:**

Correlation between almost all the scores was significant in all the diagnostic subgroups; the ROC curves of the two tests were not statistically different. A TRS of the FWT with a cut-off of ≤9/10 could accurately discriminate AD patients (sensitivity: 97%, specificity: 94%) and MCI due to AD (sensitivity: 76%, specificity: 68%) from control matches.

**Conclusion:**

FWT is a simple and valid test of hippocampal memory which appears recommendable in routine clinical practice.

## 1. Introduction

More than 1 million of individuals affected by dementia—including 600,000 subjects diagnosed with Alzheimer's disease (AD)—live in Italy today [1]; although the incidence of dementia in developed countries has been declining over the past 20 years, despite population aging [2–4], dementia care is and will remain a challenge [5] and is actually receiving increasing attention from governments and policy makers [2].

In 2011, the prevalence of dementia in our local health centre was 6.8% among people aged ≥65 [6]; in the same area, during a 10-year observation period, new patients at their first visit to a memory clinic progressively showed less severe cognitive and behavioural disturbances: this reflected a more careful approach in the general public towards recognizing cognitive symptoms and the appearance of new clinical needs in patients [7].

The current implementing management of dementia care in Italy, which was organized into the first “National Dementia Plan” in 2014 [8], still demonstrates some weaknesses, despite the efforts to implement to-be-shared strategies for promoting appropriateness and quality of care and for limiting delays and fragmentation in supplying treatment and services [9].

As for the neuropsychological battery used to define mental condition, an excessive heterogeneity at present still persists. The only mandatory test suggested from the Italian Ministry of Health in 2000 was the mini mental state examination (MMSE), that is probably the most widely used screening test for cognitive impairment. Nevertheless, it may fail to differentiate patients with mild cognitive deficits from subjects in the normal range [10], while no single, nor simple, test exists to diagnose AD as yet. Considering “selective brain regional vulnerability” as the basis for the neuropsychological diagnostic approach [11, 12], and given that the earliest and most specific element of AD is a declarative memory impairment resulting from a functional disconnection of the hippocampus from associative neocortical regions [13], it would seem that a simple tool measuring hippocampal memory trace consolidation might be useful in clinical practice. A current hypothesis [14, 15] states that a memory paradigm which controls for the semantic encoding of incoming stimuli and facilitates retrieval by providing category cues could be the most effective in discriminating patients affected by mild forms of AD from healthy subjects, but a recent pertinent review [13] provided controversial results, which seems to suggest that further experimental work is required.

On this basis, the five-word test (FWT) was developed and validated in a French-speaking population [16, 17]: it has been demonstrated that this is a simple and valid test of verbal episodic memory, easy to use for the screening of AD.

The present study aims to validate, for the Italian language, the FWT for discrimination of patients affected by mild cognitive impairment (MCI) or dementia due to AD from their healthy matches. The effectiveness of the FWT was compared with that of a specific short story test [18], currently used for the diagnosis of memory impairment.

## 2. Methods

### 2.1. Participants and Setting

A retrospective, descriptive study was carried out by enrolling (from May 2015 to May 2016) Italian-speaking patients aged ≥60 years who were attending our memory clinic (in Brescia, Eastern Lombardy, Italy) for the first time, reporting new-onset progressive memory complaints. Patients underwent neuropsychological evaluation performed by a trained neuropsychologist and were assessed independently by a neurologist both for clinical examination and compilation of individual medical history. Patients were included when diagnosed with MCI or dementia due to AD following the *core clinical criteria* set out in the National Institute on Aging and Alzheimer's Association (NIA-AA) workgroup recommendations [19, 20]. These criteria are mainly based on the judgement of a clinician; they do not incorporate the routine use of laboratory tests, going towards identifying those individuals with AD pathophysiological processes as the likely primary cause of their progressive cognitive decline. However, in our sample, all the patients underwent also neuroimaging assessment (MRI or PET FDG scan), in order to increase the likelihood that the underlying disease was mainly a neurodegenerative disorder consistent with AD. FWT scores were not actually used to establish the final diagnosis.

Exclusion criteria were insufficient knowledge of the Italian language, moderately severe to severe dementia (MMSE score below 15), and other diagnoses (such as subjective memory complaint, other primary neurodegenerative disorder, or dementia subsequent to a pre-existing mental illness or physical disease).

The aim was to compare the accuracy of neuropsychological tools in discriminating affected patients from healthy matches. Control subjects were recruited at the local neuropsychological outpatients' clinic from elderly individuals needing to renew their driving licence; they had no reported cognitive complaints and no former diagnosis of cognitive impairment in medical records and may be considered representative of the aging population in our area.

Recruited subjects were then classified into three diagnostic subgroups: control subjects, MCI due to AD, and AD. Informed consent was obtained from all the participants or, when impossible, by their reference caregiver.

### 2.2. Data Collection and Neuropsychological Evaluation

Demographic data were noted down. Global cognitive abilities were assessed using the MMSE; the score was adjusted for sex, age, and education level following normative Italian data [21]. All the participants were screened for episodic verbal memory with the five-word test (see below) and the short story module [18]. Scoring of the short story requires evaluation of the ability to recall—substantially and (as far as possible) word-by-word—the details of a short write-up, immediately after listening to it and after 10 minutes. We recorded all the scores obtained at immediate and delayed testing and divided the combined total score by 2, likewise adjusted for sex, age, and education.

The cognitive test battery included the examination of visuospatial skills (clock drawing test) and attentive functions and mental flexibility (trail making test parts A and B).

### 2.3. The FWT

In order to carry out the test, a list of 5 words in their Italian translation (strainer, lemonade, grasshopper, museum, and lorry, respectively: “colino,” “limonata,” “cavalletta,” “museo,” “camion”), printed on a sheet of A4, was shown to the subjects, who were asked to read and, later on, to point and name aloud each item when the matching category cue was verbally given. In that way, effective encoding of to-be-remembered information was controlled. Then, the sheet was removed, and the subjects were requested to recall the words; when one or more words were not spontaneously remembered, a semantic category cue was given in order to stimulate the item's retrieval. An immediate recall score (IRS) was obtained by adding the number of spontaneously retrieved items to those retrieved thanks to the semantic cue. If the subjects failed again to recollect any words, the sheet would be shown and removed again until the missing items were identified and retrieved (max. 3 repetitions) to ensure the possibility to proceed with the second phase; this step had no impact on the individual IRS [16, 17]. During the subsequent 5 minutes, subjects performed some nonverbal interference tasks (clock drawing test, copying of the pentagons as part of MMSE); then, a delayed recall was proposed to the subjects making use of the same procedure as before, providing a delayed recall score (DRS: number of retrieved items at delayed free + cued recall).

The global number of recalled words during immediate free/cued and delayed free/cued recalls was noted down as total recall score (TRS), with a range from 0 to 10. According to the FWT validation paper [16], a score ≤ 9 should be a proper cut-off for discrimination of any dementia from memory subjective complaints.

### 2.4. Statistical Analysis

Data were analysed making use of IBM SPSS version 21.0 for Windows (SPSS Inc., Chicago, Illinois, USA). A descriptive analysis of clinical characteristics, expressed as dichotomous qualitative variables (prevalence rate, %) or quantitative variables (mean ± SD), was first performed; an ANOVA model was used to compare continuous variables among the three diagnostic subgroups, and chi-square test was used for the dichotomous ones. A value of *p* ≤ 0.05 associated with the test statistic was considered statistically significant.

The discriminative performance of the total score of the FWT was studied performing a multivariate logistic regression to account for age, education level, and sex distribution.

The Spearman rank coefficient (*ρ*), ranging from −1 to +1, was used to assess the correlation between immediate (IRS), delayed (DRS), and total score (TRS) of the FWT and the correspondent matches of the short story test.

The accuracy of the two tools in the diagnosis of MCI due to AD or AD was investigated by examining sensibility and specificity, through building receiving operator characteristic (ROC) curves.

The test reliability was assessed using intraclass coefficient correlation in a random subgroup of twenty-four subjects (10 controls, 8 MCI, and 6 AD). In particular, the inter-rater reliability between two neuropsychologists on IFR and DFR was 0.830 and 0.746, respectively.

## 3. Results

217 subjects were consecutively recruited and were subjected to all the neuropsychological tests mentioned. Demographic data of the three diagnostic subgroups (controls, MCI, and AD), together with the descriptive scores for each of the used neuropsychological parameters, are shown in Table 1.

As can be seen, control subjects differed from cognitively impaired individuals regarding sex distribution, mean age, and mean education level: they were more often males and significantly older and also had a higher level of schooling. However, the differences were bypassed (when analysing cognitive test implications) with the use of adjusted scores, whenever possible. Each subgroup statistically differed from the others in all the mean scorings of the neuropsychological instruments: as expected, MCI and AD patients performed progressively more poorly (*p* ≤ 0.001) when compared to control subjects, in almost all the cognitive tests.

Considering that data for standardized raw score adjustment are not available for the FWT [16, 17, 22, 23], the multivariate logistic regression analysis showed that the TRS remained discriminant even when taking into account age, education level, and sex distribution, both in MCI (95% CI: 0.14–0.45; *p* < 0.001) and in AD patients (95% CI: 0.004–0.18; *p* < 0.001).

The Spearman rank correlation between TRS of the FWT and the total score of the short story test was significant (*p* = 0.04 in the control subgroup; *p* = 0.001 in both the two cognitive impaired subgroups). The Spearman rank correlation between DRS of the FWT and delayed recall score was also significant in all the subgroups (*p* < 0.05). Nevertheless, the correlation between immediate recall scores of the two tools was not significant in the MCI subgroup, but was found significant in control and AD subgroups. The *ρ* value ranged from 0.20 (between the TRS of the FWT and the total score of the short story test in control subjects) to 0.60 (between the DRS of the FWT and the delayed recall score of the short story test in MCI subjects).

**Figure 1:**
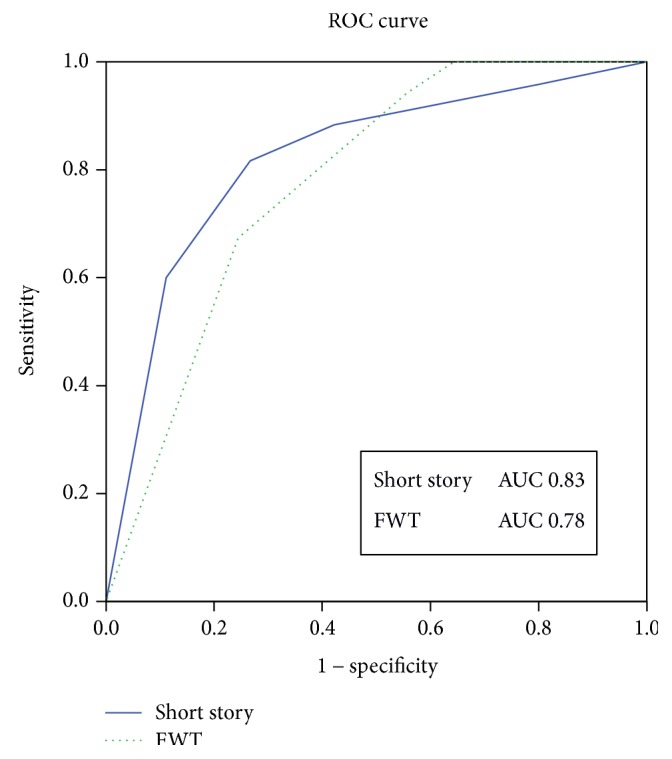
Receiving operating characteristic (ROC) curves of the short story equivalent score and the five-word test (FWT) total score: discrimination of mild cognitive impairment due to Alzheimer's disease from healthy subjects. AUC indicates the area under the curve.

**Figure 2:**
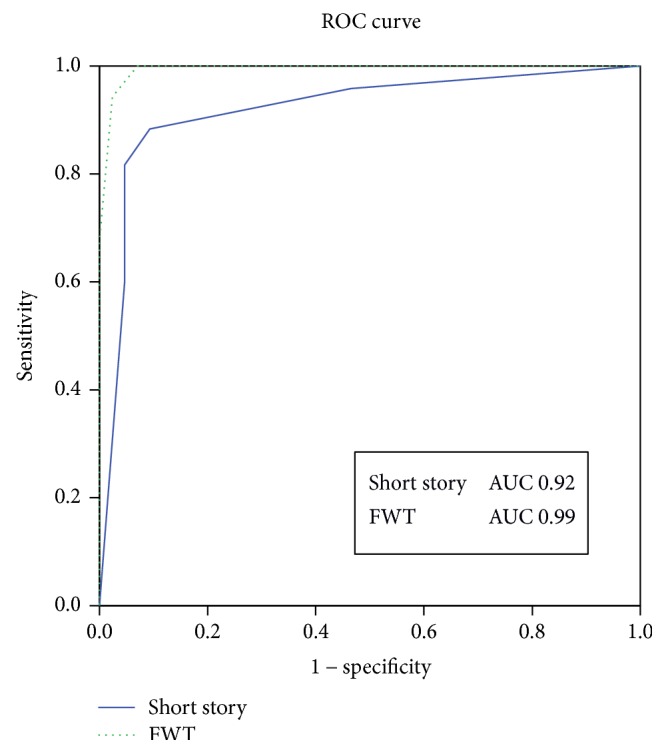
Receiving operating characteristic (ROC) curves of the short story equivalent score and the five-word test (FWT) total score: discrimination of Alzheimer's disease from healthy subjects. AUC indicates the area under the curve.

We calculated the ROC curves for the TRS of the FWT and the equivalent score (adjusted for sex and age) of the short story, to evaluate the area under the curve (AUC) for discrimination of MCI patients and of AD patients from control subjects. The AUC for MCI patients' discrimination was 0.78 for the FWT and 0.82 for the short story, while it was 0.99 and 0.92, respectively, when analysing AD patients' discrimination (Figures 1 and 2).

Considering a cut-off value of ≤9 of the TRS, the sensitivity and specificity for detecting MCI were 76% and 68%, respectively, whereas they were 97% and 94% for AD detection. On the other hand, considering a cut-off value of ≤2 of the equivalent score short story (0–4), the sensitivity and specificity for detecting MCI were 73% and 82%, respectively, whereas they were 97% and 94% for AD detection.

## 4. Discussion

Given that verbal episodic memory deficits occur in most cases of dementia of organic origin, a *core clinical* feature of MCI patients who will likely convert to dementia due to AD is an impairment of “hippocampal” memory [24], characterised by a deficit in memory trace consolidation, and not in elaborative encoding, nor in information retrieval strategies.

We investigated the construct validity of the Italian version of the FWT [16] as a measure of verbal episodic memory, and in distinguishing MCI and AD patients from controls, when compared to a short story test [18]. The latter is commonly used in our setting to measure a patient's abilities to learn verbal information and recall it after a five-minute delay. In 2012, the FWT was compared favourably with the free and cued selective reminding test (FCSRT), which is at present the gold standard for measurement of memory impairment in dementia patients [17]: this test is conceptually similar to FWT, since it is also derived from the Grober and Buschke paradigm [14], but is longer and more laborious in terms of routine daily testing.

We administered FWT and the short story test to patients suffering from MCI or dementia due to AD [19, 20] and to a control group: our findings confirm literature data [16, 17, 22] demonstrating the effectiveness of FWT as a tool in identifying organic disorders of memory encoding and in distinguishing AD patients from controls. The distribution of subjects by age, gender, and education level was different among the diagnostic subgroups, reflecting the day-to-day work in a memory clinic with a heterogeneous, unselected population. Even though raw score adjustment is not available for FWT, the test remained discriminant even when taking into account these parameters.

A significant correlation has been shown in all the subgroups between scores of the FWT and the short story test, whether in immediate or delayed or total recall. The only exception was the absence of significant correlation between the immediate scores in the MCI subgroup: this may be due to the varying level of severity of impairment (single domain versus multiple domain) among patients diagnosed with amnestic MCI due to AD [19].

The ROC curves did not differ statistically between the FWT and the short story test in identifying both MCI and AD patients, suggesting both similar accuracy and the validity of combined standardized use in routine practice.

The FWT appears to be particularly accurate in distinguishing AD patients from controls, strengthening the literature data: sensitivity (97%) and specificity (94%) were similar to that reported by Mormont et al. in their validation study [17]. However, accuracy of the FWT for amnestic MCI patients' detection has been investigated here for the first time. The varying level of severity among patients in the MCI subgroup (single versus multiple domain) may also explain the lower accuracy level of the FWT for MCI detection in our study, together with the heterogeneity of the study sample.

The current study may be limited by the fact that MCI and AD diagnoses were based on *core clinical criteria* supported only by topographical biomarkers (MRI or PET FDG). Even though the diagnosis of AD can be also improved by the use of biological measures reflecting AD pathology, the validation of their clinical usefulness and the development of standardized guidelines are still incomplete [25]. A long-term follow-up, with data of conversion to AD of the MCI patients, might be therefore useful to reinforce our results.

The FWT seems though a simple but powerful clinical instrument, designed in such a way as to target hippocampus recruitment for successful processing. As it is derived from the Grober and Buschke paradigm [14], it controls for semantic encoding of incoming stimuli and facilitates retrieval through semantic cues. In an exhaustive paper [13], a paradigm of this kind was recommended as a target for further investigation in its diagnostic accuracy for early AD detection, in order to potentially confirm the assumption that it could represent the most effective diagnostic procedure for this purpose.

## 5. Conclusion

Our study has shown that the FWT is an accurate and useful neuropsychological instrument, notwithstanding its simplicity. The rapidity of administration constitutes an advantage over the gold standard test for “hippocampal memory deficit” detection (i.e., the FCSRT), making the FWT an effective and proven resource in routine practice for memory clinic healthcare staff and general practitioners. When combined with other neuropsychological tools (e.g., MMSE, short story test), it can certainly help in generating a solid diagnostic hypothesis.

## Figures and Tables

**Table 1:** Demographic data and neuropsychological assessment of a sample of 217 patients consecutively recruited in an Alzheimer evaluation unit and of three subgroups with control subjects, mild cognitive impairment due to Alzheimer's disease (MCI), and dementia due to Alzheimer's disease (AD).

	Controls (*N* = 127)			MCI (*N* = 47)			AD (*N* = 43)			
	Mean	SD	%	Mean	SD	%	Mean	SD	%	*p*
Gender (m)			78.7^∗^^,^°			53.2^∗^^,§^			39.5°^,§^	0.000
Age (yrs)	80.0^∗^^,^°	6.2		74.9^∗^	5.6		76.2°	6.7		<0.005
Education (yrs)	8.2°	4.2		7.6^§^	3.9		5.3°^,§^	1.6		<0.01
MMSE^a^	29.1^∗^^,^°	0.8		25.7^∗^^,§^	1.6		20.2°^,§^	2.8		0.000
Clock drawing	8.9^∗^^,^°	1.4		6.9^∗^^,§^	2.2		5.2°^,§^	2.6		0.000
TMT-A	29.6°	17.7		62.8^§^	91.4		127.3°^,§^	168.9		<0.01
TMT-B	127.8^∗^^,^°	174.0		350.5^∗^	209.7		469.7°	130.6		0.000
Short story (immediate)	10.7^∗^^,^°	4.5		7.1^∗^^,§^	3.5		3.7°^,§^	2.4		0.000
Short story (delayed)	13.8^∗^^,^°	9.1		8.3^∗^^,§^	4.0		2.9°^,§^	3.0		<0.005
Short story (total)^a^	15.4^∗^^,^°	4.2		10.3^∗^^,§^	3.4		7.5°^,§^	3.8		<0.005
FWT (IRS)	4.9^∗^^,^°	0.6		4.3^∗^^,§^	0.8		3.2°^,§^	1.3		0.000
FWT (DRS)	4.8^∗^^,^°	0.4		3.8^∗^^,§^	1.4		2.0°^,§^	1.3		0.000
FWT (FRS)	8.4^∗^^,^°	1.6		5.4^∗^^,§^	2.4		3.0°^,§^	1.6		0.000
FWT (TRS)	9.6^∗^^,^°	0.6		8.1^∗^^,§^	1.9		5.3°^,§^	1.9		0.000

MMSE: mini mental state examination; TMT-A/B: trail making test—part A/B; FWT: five-word test; IRS: immediate recall score; DRS: delayed recall score; FRS: free recall score; TRS: total recall score. ^a^The table shows the scores adjusted for gender, age, and education level. Statistically different parameters: ^∗^controls versus MCI, °controls versus AD, and ^§^MCI versus AD.
